# Cyclin-Dependent 4/6 Kinase Inhibitors for Treatment of HER2-Positive Breast Cancer: 2026 Update

**DOI:** 10.3390/cancers18030533

**Published:** 2026-02-06

**Authors:** Ciara C. O’Sullivan

**Affiliations:** Department of Medical Oncology, Mayo Clinic, 200 1st Street SW, Rochester, MN 55905, USA; osullivan.ciara@mayo.edu; Tel.: +1-507-293-0526

**Keywords:** breast cancer, cyclin-dependent kinase 4/6 inhibitors, CDK4/6i, therapy

## Abstract

Cyclin-dependent kinase 4/6 inhibitors (CDK4/6i; palbociclib, ribociclib, abemaciclib, dalpiciclib) combined with endocrine therapy (ET) were a major advance in the treatment of hormone receptor-positive (HR+), human epidermal growth factor receptor 2-negative (HER2-) metastatic breast cancer (MBC) worldwide. Notably, clinical activity has also been observed in HR+HER2-positive (HER2+) MBC, with significant progression-free survival (PFS) benefits. Cyclin-dependent kinases 4/6 (CDK4/6) are downstream of HER2 and pathways driving resistance to HER2-targeted therapies. However, clinical development of CDK4/6i in HER2+ MBC slowed, given the advent of highly effective tyrosine-kinase inhibitors (TKIs) (i.e., tucatinib) and antibody–drug conjugates (ADCs) (i.e., trastuzumab deruxtecan), which currently dominate the treatment armamentarium. The observation that luminal disease defined by a predictive analysis of microarray 50 (PAM50) was independently associated with a significantly longer PFS versus nonluminal disease was important, with researchers inferring that intrinsic molecular subtypes could be used to identify patients most suitable for ET + CDK4/6i + HER2-targeted treatment. Subsequently, the phase III PATINA trial (which included patients with 1L HR+HER2+ MBC, treated with palbociclib vs. placebo with maintenance ET+ H[P]) noted a striking PFS improvement of >15 months in the palbociclib arm, renewing interest in CDK4/6i-based treatments for HR+HER2+ MBC. Herein, we review the development of CDK4/6i in HER2+ BC, discussing current challenges and potential future directions.

## Simple Summary

CDK4/6i (palbociclib, abemaciclib, ribociclib, dalpiciclib) have activity in HR+ HER2+ metastatic breast cancer (MBC). Toxicities are well described, and include neutropenia, transaminitis, long QTc (ribociclib, dalpiciclib), or nausea and diarrhea (abemaciclib). CDK4/6i penetrate the blood–brain barrier, and information is emerging regarding their therapeutic role in brain metastases (BMs). In treatment-naïve and pretreated HR+ HER2+ MBC, CDK4/6i-based regimens improve PFS vs. selected standard-of-care HER2-targeted regimens. Intrinsic molecular subtypes (PAM50 analysis) may assist with patient selection for ET+ CDK4/6i + HER2-targeted treatment.

## 1. Introduction

Internationally, 2.3 million new BC diagnoses and 670,000 BC-related deaths (in females) occurred in 2022; therefore, improved treatment options are urgently needed [1]. The classification of BC is based on molecular and histopathological characteristics [2,3]. Specifically, the majority of BCs are hormone receptor (HR)-positive (>75%), i.e., tumor expression of the estrogen receptor (ER) and/or progesterone receptor (PR) is ≥1%. Approximately 15–20% of BCs overexpress human epidermal growth factor receptor 2 [HER2]. More recently, ≥4 intrinsic subtypes of BC have been described, which can further inform treatment selection and patient prognosis [4,5]. Luminal A and B BCs express ER and/or PR, whereas luminal B BCs may have less HR expression, but higher rates of cellular proliferation, and/or HER2 overexpression. Generally, HER2-enriched BCs are ER- and PR-negative and HER2-positive. Basal-like tumors are usually triple-negative, i.e., negative for ER, PR, and HER2 (i.e., 0, 1+ by IHC or IHC 2+ and HER2 non-amplified), and are biologically aggressive (~15–20% of all BC).

Similar to triple-negative BC, HER2+ BC was historically associated with aggressive tumor behavior and inferior survival outcomes. However, seminal trials demonstrated that chemotherapy + trastuzumab dramatically improve outcomes in early and advanced HER2+ BC [6,7]. Currently, there are eight Food and Drug Administration (FDA)-approved HER2-targeted agents, but despite major progress, 16–22% of patients with HER2+ EBC and ~22–25% with MBC display primary or secondary resistance to HER2-directed therapies, ultimately dying of the disease [8]. Studying mechanisms of resistance and identifying predictive biomarkers are imperative, as HER2 status is the only approved biomarker of treatment response [9,10]. Further, an improved OS has been accompanied by an increased incidence of brain metastases (BMs), a major unmet clinical need [11]. Therefore, identifying novel systemic therapies that effectively penetrate the blood–brain barrier (BBB) and treat extracranial disease is essential [12]. There is interest in harnessing the immune system to optimize outcomes in HER2+ MBC as immune enrichment may improve responses to HER2-directed therapies [13]. A retrospective analysis of patients with newly diagnosed HER2+ MBC showed that higher levels (>20%) of tumor-infiltrating lymphocytes (TILs) in pretreatment tumor samples were associated with improved overall survival (OS) [14]. TILs may mediate disease progression via ER signaling activation, as well as having estrogen-mediated effects in other immune cell subpopulations [15].

## 2. HR+ HER2+ MBC: A Heterogenous Treatment Landscape

Approximately 10% of BCs in the United States are HR+HER2+; whether patients should be treated differently is unclear [16]. Substantial heterogeneity and variations in molecular characteristics (e.g., ER and PR expression levels, HER2 overexpression, presence of other mutations) pose challenges regarding treatment optimization, as primary oncogenic drivers differ. Further, HR−HER2+ BC is often HER2-enriched by intrinsic subtyping, but many HR+HER2+ BCs are classified as luminal A/B, suggesting a different disease biology [17]. Varying sensitivity to HER2 vs. HR pathway targeting may sometimes require targeting both to maximize the clinical benefit [18]. In HER2+ MBC, ET is usually prescribed for patients unsuitable for, or who decline, chemotherapy, or as post-chemotherapy 1L maintenance with trastuzumab ± pertuzumab. Dual blockade of HR and HER2 may overcome therapeutic resistance, reduce chemotherapy use, provide safe and effective treatment, and improve OS [16,19]. Therefore, targeting ER in HR+HER2+ BC is potentially an underutilized therapeutic strategy, with considerable benefits for selected patients. Other drugs, such as novel ET, antibody–drug conjugates (ADCs), phosphatidylinositol 3-kinase–protein kinase B (AKT)–mammalian target of rapamycin inhibitors, and checkpoint inhibitors could be further leveraged in this space. Research is ongoing to optimize patient selection by predictive biomarker discovery and use of intrinsic molecular subtypes.

## 3. HER2+ MBC—Current Treatment Paradigms

Presently, a standard treatment for first-line (1L) HER2+ MBC is a taxane (T), trastuzumab (H) + pertuzumab (P), based on CLEOPATRA (NCT00567190), which showed a 16-month OS advantage favoring THP vs. TH + placebo [20]. Based on a vastly superior PFS (~22 months) favoring the ADC, trastuzumab deruxtecan [T-DXd], in Destiny-Breast 03, this drug replaced T-DM1 as the standard second-line (2L) treatment [21]. Tucatinib, capecitabine + trastuzumab is another 2L option, given the progression-free survival (PFS) and OS advantage seen in the tucatinib arm of HER2CLIMB, including the ~50% of participants with BM at enrollment [22]. Third-line (3L) options are tucatinib, capecitabine + trastuzumab [if not previously administered], or T-DM1, and there are multiple options in the 4L and beyond [8].

An evolution in 1L therapy for HER2+ MBC is underway. Results from Destiny-Breast 09 (NCT04784715) showed that T-DXd + P improved the mPFS by ~14 months compared with standard THP [23], which led to FDA approval of T-DXd ± P in the 1L in 2025. Results from HER2CLIMB05 (NCT05132582) recently showed that the median PFS was significantly improved by 8.6 months with the addition of tucatinib versus a placebo [hazard ratio = 0.641; *p* < 0.0001] [24]. Additional 1L trials are evaluating whether other targeted therapies (i.e., phosphatidylinositol 3-kinase [PI3K] pathway inhibitors [alpelisib and inavolisib] and selective estrogen receptor downregulators [SERDs] [giredestrant]) should be added to maintenance H(P) ± ET after induction chemotherapy and HER2-directed therapy. Notably, PATINA (AFT 38/NCT02947685) showed an impressive 15-month PFS advantage in patients with HR+HER2+ MBC who received maintenance H(P) + ET + palbociclib vs. H(P) + ET alone in the 1L [25]. Herein, we review the clinical development of CDK4/6i in HER2+ BC to date, including future research directions.

## 4. CDK4/6i—Mechanism of Action and Clinical Development in HER2- BC

CDK4/6i + ET were a major treatment breakthrough in early and advanced ER+ HER2- BC [26]. CDK4/6i have a key role in proliferation by controlling G1 to S-phase cell cycle transition. Cyclin-dependent kinases (CDKs) 4 and 6 are primary targets, disrupting the CDK4/6-D-type-Rb pathway, leading to G1 cell-cycle arrest [27]. Notably, CDK4/6 or related pathway components are also overexpressed or dysregulated in different BC subtypes [28,29]. CDK4/6i also promote antitumor immunity by inducing tumor antigen presentation and clearance of tumor cells governed by T cells [30], increasing immune infiltration and inducing T-cell activation [31]. Four selective orally administered CDK4/6i are approved for clinical use. Palbociclib, ribociclib, and abemaciclib (+ET) are all approved by the FDA for 1L and 2L treatment of ER+ HER2− MBC; dalpiciclib (+ET) is approved in China for the same indications [32,33,34,35,36,37,38,39]. Abemaciclib is also approved for monotherapy of pretreated ER+, HER2− MBC. CDK4/6is + ET are most effective in patients with HR+ BC, all significantly improving PFS in HR+HER2- MBC; ribociclib and abemaciclib [+ET] also improve OS [38,40,41,42,43,44]. Ribociclib and abemaciclib (+ET) improve invasive-disease survival (iDFS) and OS (abemaciclib) [45] in patients with high-risk early-stage HR+HER2- BC [46]. Toxicities (generally manageable) include neutropenia (all), transaminitis, prolonged QTc (ribociclib), and nausea and diarrhea (abemaciclib). Abemaciclib’s activity as a monotherapy is possibly due to more potent CDK4 inhibition; its different toxicity profile (less neutropenia; more nausea and diarrhea) may also be due to greater selectivity for CDK4 than CDK6 [26].

## 5. CDK4/6i in HER2+ BC

**Figure 1:**
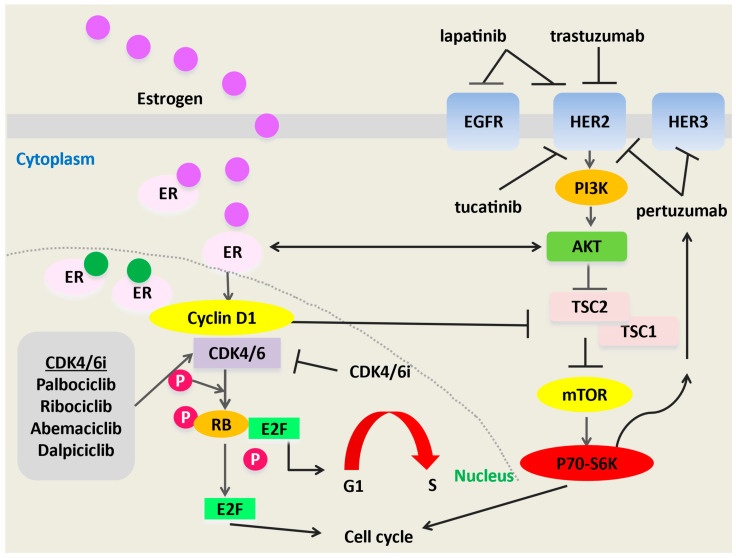
The cyclin D1-CDK4/6 axis and associated pathways. Interactions within the cyclinD1/CDK4/6 axis result in increased phosphorylation of TSC1 and TSC2, along with phosphorylation of mTOR substrates, S6K1 and 4E-BP1. Via Akt, EGFR/HER2 triggers TSC2 phosphorylation. The combination of CDK4/6i and HER2-targeted agents may more powerfully suppress mTORC1 activity compared with either drug class alone. More profound inhibition of mTORC1 activity may cancel feedback inhibition of EGFR kinases. Consequently, HER2+ breast cancer cells may become more susceptible to inhibition of EGFR/HER2. CDK4/6, cyclin-dependent kinase 4/6; CDK4/6i, CDK4/6 inhibitors; ER, estrogen receptor; EGFR, epithelial growth factor receptor; HER2, human epidermal growth factor receptor 2; mTOR, mammalian target of rapamycin; P, phosphate; Rb, retinoblastoma; TSC, tuberous sclerosis complex.

It has been known that CDK4/6i are active in both HR+HER2- and HR+HER2+ BC for >15 years [47]. CDK4/6 are downstream of HER2 and pathways driving resistance to HER2-targeted therapies (Figure 1). Studies combining CDK4/6i with HER2-directed systemic therapy in pretreated HR+HER2+ MBC showed modest PFS benefits, but were largely eclipsed by approval of the novel TKI, tucatinib, and the ADC, trastuzumab deruxtecan (T-DXd), both highly effective treatments for both HR+ and HR-HER2+ MBC. Following FDA approval of T-DXd for 2L treatment of HER2+ MBC [21], several trials evaluating CDK4/6i + HER2-directed therapies were halted. However, trials evaluating dalpiciclib in combination with HER2-directed therapies continued in China, where T-DXd was not approved until 2023. Results from PATINA (NCT02947685), an FDA Registration trial launched in 2017, which evaluated HER2-directed therapy + ET ± palbociclib as 1L treatment for HR+HER2+ MBC after completion of induction chemotherapy and HER2-directed therapy, were presented in 2024 [25]. This was a historic trial, noting an impressive 15-month PFS improvement favoring the palbociclib arm. Therefore, a renewed interest in CDK4/6i for treatment of HR+HER2+ MBC globally has arisen. A major question is whether select patients may enjoy excellent oncologic outcomes and minimal toxicities on CDK4/6i, ET, and HER2-directed therapies alone.

## 6. CDK4/6i in HER2+ BC: Preclinical Data

The relevance of the CDK4/6-D-type-Rb pathway in HER2+ cell lines was demonstrated >20 years ago [27]. Specifically, mammary tumors displayed high levels of cyclin D1 protein, given amplification of wild-type or activating Neu mutations in MCF cells and transgenic mice [48]. The extracellular region of C-terminal autophosphorylation sites on MCF7 cells with activating Neu mutations plays an important role in cyclin D1 activation. The transcription factor E2F1 likely initiates robust HER2 signaling to cyclin D1, a critical downstream target of Neu-induced transformation. HER2 pathway signaling also induces carcinogenesis by enabling E2F1-driven DNA metabolism and replication genes, in addition to phosphorylating and activating a transcriptional coactivator, SRC-3 [49]. Following the discovery of a CDK-signaling node, it was deduced that palbociclib combined with the TKI lapatinib suppressed de novo DNA synthesis, largely via disruption of E2F1 and target genes. Other observations were that luminal ER+ BC cell lines (including those overexpressing HER2) were sensitive to palbociclib while other subtypes were resistant [50]. Palbociclib also synergized with tamoxifen and trastuzumab in ER+ and HER2+ cell lines, respectively [27]. In an ex vivo model of breast tumor tissue evaluating the cytostatic response to palbociclib [51], inhibition of cellular proliferation was seen in ~85%, regardless of ER or HER2 status. Other preclinical studies demonstrated that palbociclib inhibited proliferation in resistant and nonresistant HER2+ cell lines [52], and that CDK4/6i may induce T-cell activation and enhance T-cell activity [30]. CDK4/6i also penetrate the BBB with varying efficacy [53,54]. Overall, preclinical data supported the clinical investigation of CDK4/6i, mainly in HR+HER2+ MBC. More recently, tucatinib + palbociclib + fulvestrant was investigated in HR+HER2+ BC cell lines. Tucatinib + fulvestrant and tucatinib + palbociclib doublets were synergistic; adding fulvestrant to tucatinib + palbociclib further improved efficacy in all three cell lines [55].

## 7. Resistance/Response to HER2-Directed Therapies and the Cyclin D1/CDK4/6/pRb Axis

Primary and secondary resistance mechanisms to HER2-targeted agents have been well described by others [56,57]. Examples include alterations in the phosphoinositide-3 kinase [PI3K-Akt] and phosphatase and tensin homolog (*PTEN*) pathways [58], increased epithelial growth factor receptor (*EGFR*) and insulin-like growth factor levels, and crosstalk between mammalian target of rapamycin (mTOR), PI3K, and mitogen-activated protein kinase signaling pathways [59].The cyclin D1/CDK4/6/pRb axis can also promote resistance to HER2-directed treatment (Figure 1) [60,61]. Additionally, interactions between HER2 and the PI3K-Akt pathway may trigger cyclin D1 activation downstream, ultimately leading to therapeutic resistance. In a transgenic HER2+ BC mouse model [62], malignant cells resistant to HER2-directed therapy were noted to have elevated levels of nuclear cyclin D1 and CDK4. Inhibition of both cyclin D1 and CDK4 was synergistic, highlighting their vital role in acquired resistance to HER2-targeted therapies. Further, cell lines resistant to HER2-directed treatments were resensitized with the addition of CDK4/6i [62]. The anticancer properties of abemaciclib were explained by its ability to restore EGFR kinase family signaling by downstream Akt kinase. Dysregulation of these immune pathways is implicated in CDK4/6i resistance.

Regarding predictors of resistance to CDK4/6is, numerous mechanisms have been described, including Rb protein loss, Cyclin E/CDK2 amplification, CDK6 upregulation, PI3K/Akt/mTOR pathway activation, Aurora Kinase A overexpression, and *PTEN* loss [63,64,65,66,67,68]. Efforts to discover reliable biomarkers of CDK4/6i response and resistance are ongoing [64,66]. In HER2+ MBC, generally, promising biomarkers include HER2 levels and heterogeneity, HER3, intrinsic molecular subtypes (PAM50 analysis), DNA mutations [*PIK3CA*, *BRCA1/2*, *ERBB2*], and immune-related factors (programmed death-ligand 1 [PD-L1], TILs, FcɤR) [69,70].

## 8. CDK4/6i in HER2+EBC: Results from Selected [Neo]Adjuvant Trials

NA-PHER2 (NCT02530424) was a phase II neoadjuvant trial evaluating palbociclib, trastuzumab, and pertuzumab + fulvestrant in HR+HER2+ EBC. A co-primary endpoint was the change in baseline Ki67 expression, measured 2 weeks after treatment initiation and at breast cancer surgery. Notable secondary endpoints were the clinical objective response and pathological complete response (pCR) to neoadjuvant systemic therapy. A significant decrease in both Ki67 levels and apoptosis was noted after 2 weeks on systemic therapy, and at definitive surgery. Overall, this regimen significantly reduced Ki67 levels 2 weeks after treatment initiation, and at breast cancer surgery [71]. Eight of thirty evaluable patients (27%) had a pCR. Another phase II neoadjuvant trial, PALTAN [NCT02907918], studied palbociclib + letrozole + trastuzumab in HR+HER2+ EBC. Treatment was well tolerated, but the trial terminated early due to futility (pCR rate 7.7%) [72]. Ki67 data and RNA sequencing displayed potent anti-proliferative effects of the regimen, despite significant variability of intrinsic subtypes. Although pCR rates are generally lower in ER+ vs. ER- BC following preoperative chemotherapy and HER2-directed therapy [73], the low pCR rates observed in PALTAN and other trials suggests that chemotherapy is important for some of these patients.

eMonarcHER (NCT047523320) was a randomized, double blind, placebo-controlled phase III study (N = 2450) of abemaciclib + adjuvant ET in high-risk, node-positive, HR+HER2+ EBC post completion of adjuvant HER2-directed therapy. The study was terminated due to enrollment challenges and the evolving treatment landscape. Other neoadjuvant CDK4/6i trials in HER2+ EBC (completed and ongoing) are shown in Table 1 and Table 2.

### 8.1. CDK4/6i in HER2+ MBC: Selected Trials

Table 1 and Table 2 show completed and ongoing trials of CDK4/6i-based regimens in HER2+ MBC.

### 8.2. Phase I

Abemaciclib monotherapy was evaluated in a phase I study of patients with advanced solid tumors; activity was noted in patients with advanced ER+HER2+ BC [83]. Among the 11 patients with HR+HER2+ MBC, 4 (36%) partial responses were observed. Frequent adverse events (AEs) among the 36 patients with HR+MBC were grade 1 or 2 diarrhea. One patient (5%) developed grade 3 diarrhea; however, there were no associated treatment discontinuations. Six patients (32%) had documented grade 3 neutropenia.

The phase 1/2 ASPIRE trial (NCT03304080) evaluated ET (anastrozole) + palbociclib and trastuzumab + pertuzumab as 1L treatment for HR+HER2+ MBC [28]. The median PFS was 21.2 months (95% CI, 18.4–57.2); median OS had not yet been reached. Results were presented in late 2023 and were an interesting prelude to the 2024 results from PATINA.

Another phase IB/II trial evaluated an oral triplet regimen, tucatinib + letrozole + palbociclib (TLP) in HR+HER2+ MBC [76]. Patients had received 2L+ of HER2-directed therapy in any setting and ≤2 lines of ET for metastatic disease. Median PFS was 8.4 months in 40 evaluable patients, with an acceptable safety profile. TLP could eventually be a reasonable option for HR+HER2+ MBC, as maintenance or primary therapy for selected patients.

### 8.3. Phase II

PATRICIA (NCT02448420) randomized patients with HER2+ MBC pretreated with 2–4 lines of HER2-directed therapy to palbociclib + trastuzumab ± ET (letrozole) [74]. Six-month PFSs in patients with HR+HER2+ MBC who concomitantly received letrozole were 46.4% and 42.9%, respectively, suggesting that CDK4/6i + ET+ HER2-targeted therapy was active in this setting. Furthermore, luminal disease defined by PAM50 was independently associated with a longer median PFS vs. non-luminal disease (10.6 vs. 4.2 months, respectively; hazard ratio 0.40; *p* = 0.003), reinforcing that patients with luminal HER2+ MBC may benefit most from CDK4/6i.

Based on results from PATRICIA cohorts A and B, enrollment was discontinued, and cohort C was recruited and randomized 1:1 to evaluate the efficacy of palbociclib, trastuzumab + ET vs. the physician’s treatment of choice (TPC) in luminal A/B ER+HER2+ MBC [84]. Patients who received ≥1 prior line of HER2-targeted treatment were included. The primary endpoint was PFS. The trial recruited 73 of a planned 102 participants. Palbociclib + trastuzumab + ET significantly improved PFS vs. TPC (9.1 vs. 7.5 months), with an overall response rate (ORR) of 18.9% [95% CI 8.6–35.7] in the palbociclib arm and 7.5% [95% CI 1.4–28.5] in the TPC arm. Although these results were clinically and statistically significant, the trial was underpowered; therefore, further studies are needed.

In MONARCHER (NCT02675231), participants with HR+HER2+ MBC who had received ≥2 HER2-targeted therapies underwent randomization to one of three treatment groups: A] abemaciclib, trastuzumab + fulvestrant, B] abemaciclib + trastuzumab, and C] trastuzumab + physicians’ choice of chemotherapy [79]. The median PFS was 2.6 months longer in patients who received Arm A vs. Arm C treatment (8.3 vs. 5.7 mos. *p* = 0.051). In exploratory RNA-seq analyses, patients with luminal A/B disease subtypes were noted to have a significantly longer PFS and OS vs. those with non-luminal MBC: PFS 8.6 vs. 5.4 mos. (HR, 0.54; 95% CI, 0.38–0.79) and OS (31.7 vs. 19.7 months [HR, 0.68; 95% CI, 0.46–1.00]). Therefore, the chemotherapy-free regimen significantly improved PFS and OS vs. chemotherapy + trastuzumab. However, in 2022, clinical development of abemaciclib in HER2+ BC was halted given the evolving therapeutic landscape (FDA approvals of T-DXd and tucatinib-based therapies).

DAP-Her-01 (NCT04293276) assessed the efficacy and safety of dalpiciclib + the TKI pyrotinib as a 1L treatment for HER2+ MBC [85]. At the 25.9-month median follow-up, the ORR was 70% (95% CI: 53.5–83.4%), mPFS was 11.0 months (95% CI: 7.3–19.3 months), and OS data were awaited. Therefore, dalpiciclib + pyrotinib is a potential treatment option for HER2+ MBC patients ± BM in China. A follow-up trial, DAP-HER-02 (NCT05328440), is ongoing.

### 8.4. Phase III

DETECT V (NCT02344472) was developed to evaluate the role of induction chemotherapy in HR+HER2+ MBC [86]. Overall, 271 patients were randomized to ET + HP ± chemotherapy. Study treatment was 1L therapy for MBC in 75% of participants. The trial was later amended to include ribociclib in both arms. At the second interim analysis, the PFS and OS between treatment arms were comparable, regardless of whether or not chemotherapy was administered. However, in patients who received ribociclib, a statistically significant PFS (HR: 0.57; 95% CI: 0.39–0.85) and OS (HR: 0.47; 95% CI: 0.26–0.85) benefit was observed in a post hoc analysis of sequentially treated populations. These results are in line with PATINA and earlier studies, highlighting the clinically relevant activity of CDK4/6i in luminal B/HER2 disease [81].

PATINA is a pivotal, open-label, international randomized study that accrued patients with HR+HER2+ MBC without progression after induction therapy (6–8 cycles of taxane/vinorelbine + HER2-directed treatment); randomization was 1:1 to maintenance trastuzumab ± pertuzumab + ET ± palbociclib until disease progression [25]. ET options were an aromatase inhibitor or fulvestrant. LHRH agonists were mandatory for premenopausal women. The primary endpoint was PFS. Key secondary objectives were measures of tumor control, OS, safety, and quality of life (QoL). The main translational science objective is to compare PFS based on PIK3CA mutation status. mPFS was improved in the palbociclib arm at 44.3 months (95% CI: 32.4–60.9) vs. 29.1 months (95% CI: 23.3–38.6) in the control arm (HR 0.74 [95% CI, 0.58–0.94; 1-sided *p* = 0.0074]). Grade 3 neutropenia was the most frequent AE in the palbociclib arm. Grade 2/3 fatigue, stomatitis, and diarrhea were also more common. Overall safety was reassuring, and patient-reported outcomes are awaited. The landmark PFS results indicate that targeting more than the HER2 pathway is warranted in HER2+ MBC.

### 8.5. HER2+ Brain Metastases [BM] and CDK4/6i

Up to 50% of patients with HER2+ MBC develop BM [87]. Despite advances, treatments for HER2+ BM and leptomeningeal disease (LMD) are suboptimal. Further, BMs are the first site of recurrence in <10% of patients with HER2+ EBC [88]. Regarding CDK4/6i, abemaciclib reaches higher central nervous system (CNS) concentrations vs. palbociclib. Palbociclib was not active in HER2+ MBC with BM in one study [n = 12] [89]. A phase 2 trial evaluated the intracranial objective response rate (iORR) of abemaciclib in HR+ BM [90]. The trial enrolled four cohorts: HR+HER2- MBC; HR+HER2+ MBC; LMD; and patients for whom surgery was planned. Patients received abemaciclib 150 or 200 mg twice daily ± trastuzumab. Plasma and CNS concentrations of abemaciclib surpassed the requisite level for CDK4/6 inhibition. Among patients who received preoperative abemaciclib and later underwent surgery, the abemaciclib concentration in BM reached levels likely to cause cell cycle arrest. Of 58 participants who had a diagnosis of HR+HER2- BC, 3 patients had intracranial partial responses [intracranial ORR of ~5%]. The intracranial clinical benefit rate was 25% [95% CI 13.1–35.2], and the median intracranial PFS was approximately 5 months. In the HR+HER2+ MBC cohort, no confirmed intracranial responses were noted at the interim analysis; therefore, the trial was terminated. However, 12/27 were noted to have stable intracranial stable disease, which was sustained for >6 months in three participants. Among patients who had LMD (seven with HR+HER2- MBC and three with HR+HER2+ MBC), one patient had a complete response to treatment. Overall, the mPFS was 5.9 months and the median OS was 8.4 months. The survival outcomes were longer than previously reported in LMD. While the study did not meet its primary endpoint, it demonstrated that therapeutic doses of abemaciclib are achieved in BM. Notably, receipt of CDK4/6i prior to developing BM may lead to resistance mechanisms, which subsequently reduce CNS PFS and OS upon CDK4/6i rechallenge. Therefore, work investigating biomarkers for patient selection is needed [91]. Results from the CNS analysis of PATINA were encouraging, showing that the addition of palbociclib to HER2-targeted and endocrine therapy was associated with a lower incidence of CNS metastases. As palbociclib may delay or prevent CNS involvement in this setting, validation in other studies is warranted [92].

## 9. Ongoing Challenges and Future Directions

Given the results of PATINA, palbociclib + ET + maintenance trastuzumab ± pertuzumab after induction chemotherapy and HER2-directed therapy will likely become a new 1L treatment standard for HR+HER2+ MBC. An increased duration of response to maintenance therapy allows patients to avoid chemotherapy-related toxicities (alopecia, fever, and nausea). Further, delaying more toxic therapy in a largely non-curative-intent treatment population, many of whom will receive multiple therapy lines, could meaningfully improve QoL. Regarding later-line CDK4/6i-based regimens evaluated to date in HR+HER2+ MBC, advantages include increased PFS with no significant toxicity increase. Disadvantages, however, include an increased cost without significant OS advantage (despite numeric improvement in luminal subsets). Determining how to integrate CDK4/6i in HER2+ BC will be critical as interest resurges in this approach.

As T-DXd ± P is now an FDA-approved 1L option, it is unclear how we will integrate results from 1L post THP maintenance trials such as PATINA and HER2CLIMB 05 into the HER2+ MBC treatment paradigm. The phase II DEMETHER trial [NCT06172127] is exploring maintenance therapy with subcutaneous HP ± ET, after 1L T-DXd induction. If larger, randomized studies exploring this approach are successful, future trials could evaluate maintenance therapy with CDK4i + HP in ER+HER2+ MBC following T-DXd induction. Further, although T-DXd is highly effective, some patients are not candidates given medical co-morbidities or toxicity concerns (interstitial lung disease, alopecia, nausea). As palbociclib + trastuzumab + ET significantly improved PFS in pretreated PAM50 Luminal A or B HER2+ ABC vs. TPC, this regimen could also be a 2L+ option for patients with HR+HER2+ MBC if T-DXd is unsuitable. Re-evaluating how to sequence therapies in HR+HER2+ MBC may be increasingly relevant as interest resurges in the use of CDK4/6i for these patients. Harnessing intrinsic subtypes to personalize therapy for triple-positive MBC may allow us to identify opportunities to safely omit chemotherapy, for example, in older patients with oligometastatic bone ± nodal disease. Further study of non-cytotoxic regimens for the treatment of ER+HER2+ MBC including CDK4/6i are ongoing or planned.

Novel therapeutic partners are also being evaluated with CDK4/6i + HER2-directed therapy (i.e., ET and immunotherapies). Zanidatamab is a HER2-targeted bispecific antibody that simultaneously binds two non-overlapping epitopes (biparatopic binding). The unique structure and increased binding yield multiple mechanisms of action, including immune-mediated cytotoxicity [93]. In a phase 2A trial evaluating zanidatamab + palbociclib + fulvestrant in pretreated HR+HER2+ MBC (NCT04224272), PFS6 was 67% and mPFS 12 months (median four prior treatment lines). PAM50 subtyping was available for 29/51; luminal B had a numerically longer mPFS (11.7 vs. 9.3 mos.; *p* = 0.74) and similar PFS6 (66.7% vs. 62.5%). Further, DAP-HER-02 [NCT05328440] is investigating dalpiciclib + pyrotinib + fulvestrant or inetetamab (a novel HER2-targeted mAb), for 1L HER2+ MBC, based on the HR+ status. Re-evaluating how to sequence novel therapies in HR+HER2+ MBC will be increasingly relevant if the use of CDK4/6i is routinely integrated. Novel ET will likely be further evaluated in this space moving forward. Specifically, an EMBER-1 cohort (NCT04188548) studied abemaciclib + the selective estrogen receptor degrader, imlunestrant + trastuzumab ± pertuzumab, in de novo HR+ MBC. However, the short follow-up duration and small numbers prevented conclusions regarding the efficacy of the regimen in HR+HER2+ MBC. Combinations with other drugs, such as ADCs, PI3Ki, and checkpoint inhibitors, may be evaluated in HR+HER2+ BC moving forward [16]. Further research will need to identify patients who derive the optimal clinical benefit.

## 10. Summary and Conclusions

The striking benefits of adding maintenance palbociclib to ET and HER2-directed therapy in patients with 1L HER2+ MBC have important implications for the 1L treatment paradigm. Therefore, renewed efforts to integrate CDK4/6i + ET into the ER+HER2+ MBC treatment paradigm are needed. However, CDK4/6i are expensive, and increased PFS modestly in later-line studies, despite clinically significant differences in luminal disease. However, as chemotherapy-free options may be less toxic and effective for certain patients, further study is needed, especially in light of the recent approval of T-DXd ± P as a 1L treatment option. Ongoing areas for research optimization include patient selection, including the identification of predictive biomarkers. Leveraging intrinsic molecular subtypes to personalize therapy for HR+HER2+ MBC may identify clinical scenarios whereby chemotherapy can be safely reduced or omitted; biomarker results from PATINA and ongoing 2L+ trials will be informative. As up to 50% of patients with HER2+ MBC ultimately develop BM, incorporating agents with intracranial efficacy is critical with all lines of therapy, for prevention and treatment purposes. Therefore, better defining the role of CDK4/6i for the treatment of HER2+ BM is also required. Overall, treatment decisions should be guided by balancing clinically meaningful PFS and OS outcomes (including CNS activity) vs. increased therapeutic and financial toxicities.

## Figures and Tables

**Table 1:** Notable trials of CDK4/6is in HER2+ breast cancer with results.

Agent	Trial	Phase	Prior Lines of HER2+ Treatment	Treatment Regimen[s]	Results	Comments
**Palbociclib**	**PATRICIA** SOLTI-1303, NCT02448420 N = 71 [74]	II	2–4	palbociclib + H ± ET:	**PFS6**: Cohort B1: 42.8% Cohort B2: 46.4%	B1 + B2: ER+
**Palbociclib**	**NCT03530696** N = 18 [75]	I/Ib	Prior H+ taxane	palbociclib+T-DM1	**MTD**: not reached **ORR: 33%** (95% CI, 13–59) **mPFS** 6 mos. (95% CI, 18.4–57.2)	D1 T-DM1 and days 5–18 palbociclib Most common G3 toxicity: hematologic (>10%)
**Palbociclib**	**NCT03054363** N = 42 [76]	Ib/II	3L+	palbociclib + tucatinib + letrozole	**ORR**: 44.5% **mPFS**: 8.4 mos.	**CNS cohort**: 26.6% on study for ≥1 year G3 neutropenia: most frequent AE (64.3%)
**Palbociclib**	**NCT04224272** N = 51 [77]	IIa	1+	palbociclib + fulvestrant + **zanidatamab**	**PFS6**: 67% **mPFS**: 12 mos.	Median 4L prior therapy
**Palbociclib**	**NCT03304080** **ASPIRE** **N = 30** [78]	I/II	0	palbociclib + H + P + anastrozole	**CBR**: 97% (*p* < 0.0001) **ORR**: 73% (95% CI, 54–88%) **mPFS**: 21.2 mos. [95% CI, 18.4–57.2] **mOS**: not reached	**G3/4 AE**: neutropenia (46%) leukopenia (23%) anemia (17%)
**Palbociclib**	**PATINA** **AFT-38,** **NCT02947685** N~518 [25]	III	None for MBC [1L]	**Post induction THP**: Palbociclib + H(P) + ET vs. Placebo + H(P) + ET	**mPFS**: palbociclib arm: 44.3 mos. (95% CI: 32.4–60.9) vs. 29.1 mos. in H ± P + ET alone arm	G3 neutropenia most frequent AE: 63.2% (palbociclib) vs. 2.0% (H ± P + ET alone)
**Abemaciclib**	**MonarchHER** NCT02675231 N = 237 [79]	III	2+	abemaciclib + H ± fulvestrant (**Arm A**) abemaciclib + H (**Arm B**) TPC (chemo + H) (**Arm C**)	**Arm A** improved mPFS and mOS (numeric) vs. **Arm C** 8.3 vs. 5.7 mos.; *p* = 0.051	**mOS**: 31.1 mos. **[Arm A]** 29.2 mos. **[Arm B]** 20.7 mos. **[Arm C]**
**Ribociclib**	**NCT02657343** N = 13 [80]	Ib/II	2L+	3 cohorts **A**: Ribociclib D5-18; DE 300–600mg + T-DM1 D1 q21 D **B**: Ribociclib 400mg D1-21 + H q21 D **C**: Ribociclib D1-28 + H q 21d + fulvestrant	**No DLTs** 1 pt: 8.3% SD > 24 weeks **ORR**: 0% **mPFS**: 1.3 mos. (95% CI, 0.92–2.57)	67% HR+ HER2+ MBC Median 5 lines prior treatment No new safety concerns at 400 mg dose G3 AEs (33.3%) Neutropenia: n = 2 Fatigue: n = 1 Pain: n = 1
**Ribociclib**	**DETECT V** **NCT02344472** N = 262 [81]	III	HR+ HER2+ MBC 1L+	Evaluating induction THP Randomization: ET + HP ± chemo Ribociclib added to **both arms** after 120 pts enrolled	**OS + PFS**: similar with chemo vs. non chemo Adding ribociclib to HP + ET (chemo free): **mPFS**: 27.2 vs. 15.6 mos., HR 0.61, 95% CI 0.38–0.98, *p* = 0.040 **mOS**: Not reached vs. 38.7 mos., HR 0.48, 95% CI 0.24–0.94, *p* = 0.033	No new safety concerns Chemo-free treatment for HR+ HER2+ MBC is a 1L option Adding ribociclib may improve OS
**Dalpiciclib**	**DAP-HER-01** N = 41 [82]	II	≤1	dalpiciclib + pyrotinib	**cORR 70%, mPFS: 11 mos.**	Baseline BM: mPFS = 11 mos.

**Legend**: BC, breast cancer; HER2, human epidermal growth factor 2; H, Herceptin; P, Perjeta; PFS6, progression-free survival at 6 months; mPFS, median progression-free survival, CBR, clinical benefit rate; mos., months; L, line[s]; TPC, treatment of physician’s choice; mOS, median overall survival; cORR, complete overall response rate; BM, brain metastases, MBC, metastatic breast cancer; MTD, maximum tolerated dose; DLT, dose-limiting toxicity, ET, endocrine therapy; ER, estrogen receptor; N, number.

**Table 2:** Selected trials of CDK4/6i in HER2+ breast cancer that are recruiting/ongoing [no results].

Agent	NCT	Phase	Target Population	Agents in Regimen[s]	Primary Outcome Measure	Start Date and Projected Completion Date
**Palbociclib**	**NCT05969184**	I/II	HR+HER2+ MBC 1L	Letrozole/exemestane + H + P + palbociclib	PFS	12/2022 Unknown
**Ribociclib**	**NCT06481956**	Ib/II	HR+HER2+ MBC 1L postmenopausal	**IB**: letrozole + H + ribociclib (DE 200–600 mg) **II**: letrozole + H+ ribociclib RP2D	RP2D PFS	06/2019 10/2027
**Ribociclib**	**NCT05319873** Phase Ib, DE study → phase II study	Ib/II	HER2+ EBC 1L **II: HR + pts** randomized to arm A/B **II: HR-pts** randomized to arm B/C	**IB**: ribociciclib D1-21+ tucatinib+ H (weekly) **II**: **Arm A**: ribociciclib D1-21+ tucatinib+ H+ fulvestrant X 6 **Arm B**: docetaxel, carboplatin, H+ P X 6 **Arm C**: ribociciclib D1-21+ tucatinib+ H X 6	Safety RP2D pCR	04/2022 04/2027
**Dalpiciclib**	**NCT05574881**	I/II	HR+HER2+ MBC 1L	dalpiciclib + fulvestrant + H + P	PFS [6 weeks]	09/2022 01/2025
**Dalpiciclib**	**NCT03772353**	I/II	HR+HER2+ MBC 1-2L	dalpiciclib + pyrotinib + ET	AEs, ORR	05/2019 Unknown
**Dalpiciclib**	**NCT05328440** **DAP-HER-02**	II	HER2+ MBC (H-sensitive) 1L	dalpiciclib + pyrotinib + fulvestrant (ER+) OR inetetamab (ER-)	PFS	03/2022 01/2026

**Legend**: AEs, adverse events; BC, breast cancer; HER2, human epidermal growth factor 2; D, day; DE, dose escalation; ER, estrogen receptor; H, Herceptin; P, Perjeta; PFS, progression-free survival; pCR, pathologic complete response; L, line[s]; ORR, overall response rate; MBC, metastatic breast cancer; RP2D, recommended dose for phase 2 evaluation; ET, endocrine therapy; ER, estrogen receptor.
